# Diagnostic accuracy of novel serological biomarkers to detect acute mesenteric ischemia: a systematic review and meta-analysis

**DOI:** 10.1007/s11739-017-1668-y

**Published:** 2017-05-06

**Authors:** Nikki Treskes, Alexandra M. Persoon, Arthur R. H. van Zanten

**Affiliations:** 1 Department of Intensive Care Medicine, Gelderse Vallei Hospital, Willy Brandtlaan 10, 6716 RP Ede, The Netherlands; 2 Department of Surgery, St. Antonius Hospital, Koekoekslaan 1, 3435 CM Nieuwegein, The Netherlands

**Keywords:** Acute abdomen, Intestinal fatty acid-binding protein, Glutathione *S*-transferases, d-Lactate, Ischemia modified albumin, Citrulline, Biomarker, Acute mesenteric ischemia, Non-occlusive mesenteric ischemia, Diagnostic accuracy

## Abstract

Laparotomy remains the gold standard for diagnosis of acute mesenteric ischemia (AMI), but is often unhelpful or too late due to non-specific clinical and radiological signs. This systematic review and meta-analysis aims to evaluate the diagnostic accuracy of the novel serological biomarkers intestinal fatty acid-binding protein (I-FABP), α-glutathione *S*-transferase (α-GST), d-lactate, ischemia modified albumin (IMA), and citrulline to detect AMI. A systematic search of electronic databases was performed to identify all published diagnostic accuracy studies on I-FABP, α-GST, d-lactate, IMA, and citrulline. Articles were selected based on pre-defined inclusion and exclusion criteria. Risk of bias and applicability were assessed. Two-by-two contingency tables were constructed to calculate accuracy standards. Summary estimates were computed using random-effects models. The search yielded 1925 papers, 21 were included in the final analysis. Pooled sensitivity and specificity for investigated biomarkers were: I-FABP (Uden); 79.0 (95% CI 66.5–88.5) and 91.3 (87.0–94.6), I-FABP (Osaka); 75.0 (67.9–81.2) and 79.2 (76.2–82.0), d-lactate; 71.7 (58.6–82.5) and 74.2 (69.0–79.0), α-GST; 67.8 (54.2–79.5) and 84.2 (75.3–90.9), IMA; 94.7 (74.0–99.9) and 86.4 (65.1–97.1), respectively. One study investigated accuracy standards for citrulline: sensitivity 39% and specificity 100%. The novel serological biomarkers I-FABP, α-GST, IMA, and citrulline may offer improved diagnostic accuracy of acute mesenteric ischemia; however, further research is required to specify threshold values and accuracy standards for different aetiological forms.

**Electronic supplementary material:**

The online version of this article (doi:10.1007/s11739-017-1668-y) contains supplementary material, which is available to authorized users.

## Introduction

Acute mesenteric ischemia (AMI) is a rare, but potentially catastrophic medical condition with mortality rates up to 58–80% in the critical care setting [1, 2]. Various mechanisms may provoke intestinal ischemia, either from vascular or obstructive origin, such as bowel strangulation [3–5]. Four aetiological forms of vascular AMI have been identified [6]: arterial embolism, arterial thrombosis, venous thrombosis, and non-occlusive mesenteric ischemia (NOMI). NOMI may be caused by profound and disproportionate splanchnic vasoconstriction during low flow states in critically ill patients, or perioperative during major aortic surgery when splanchnic blood flow is disrupted or mesenteric arteries are sacrificed [7–9]. Early diagnosis is pivotal for reversal of ischemic damage, whereas delayed intervention may result in intestinal necrosis, multiple organ dysfunction syndrome, and death. However, diagnosis is difficult, particularly in the early stages when treatment is most beneficial [10, 11]. Performance of currently available laboratory tests is suboptimal (e.g., the l-lactate sensitivity and specificity is 86 and 44% [12]). The best diagnostic test apart from diagnostic laparotomy remains contrast computed tomography (angiography), (sensitivity 94%, specificity 95% [12, 13]). Several new biomarkers may facilitate diagnostic accuracy and will be addressed in this article.

Intestinal fatty acid-binding protein (I-FABP) is a small cytosolic protein exclusively expressed by enterocytes and is rapidly released into the circulation in case of mesenteric cell damage [14, 15]. The short lifetime of plasma I-FABP (11 min) facilitates the tracking of ischemic enterocyte damage almost in real time [16]. The glutathione *S*-transferases (GSTs) are a family of enzymes involved in intracellular detoxification. The α-subunit of GST is present in the liver and small intestines. The plasma level of α-GST has been suggested to be a sensitive marker of small bowel ischemia [17, 18]. d-Lactate is the stereoisomer of l-lactate and is produced by colonic bacteria only as a product of fermentation. Elevated d-lactate levels have been associated with bacterial overgrowth due to infection [19], short bowel syndrome [20] and mesenteric infarction [21]. Ischemia modified albumin (IMA) is human serum albumin that is less capable of binding cobalt due to ischemia [22]. Elevated IMA plasma levels have been associated with myocardial ischemia [23], but may also be of value for the diagnosis of mesenteric ischemia. IMA is measured through the cobalt–albumin-binding assay (CABA) test. Citrulline is an amino acid produced in the mitochondria of mature enterocytes. It has been shown that plasma citrulline is an accurate biomarker of the functional enterocyte mass and a plasma concentration less than 20 μmol/L is a marker of enterocyte mass reduction [24]. Its circulating half-life is 3–4 h [25, 26].

The aim of the present study is to perform a systematic review and meta-analysis of the available literature concerning the diagnostic accuracy and predictability of I-FABP, α-GST, d-lactate, IMA, and citrulline as serological biomarkers for the early diagnosis of AMI.

## Methods

### Search strategy

**Fig. 1:**
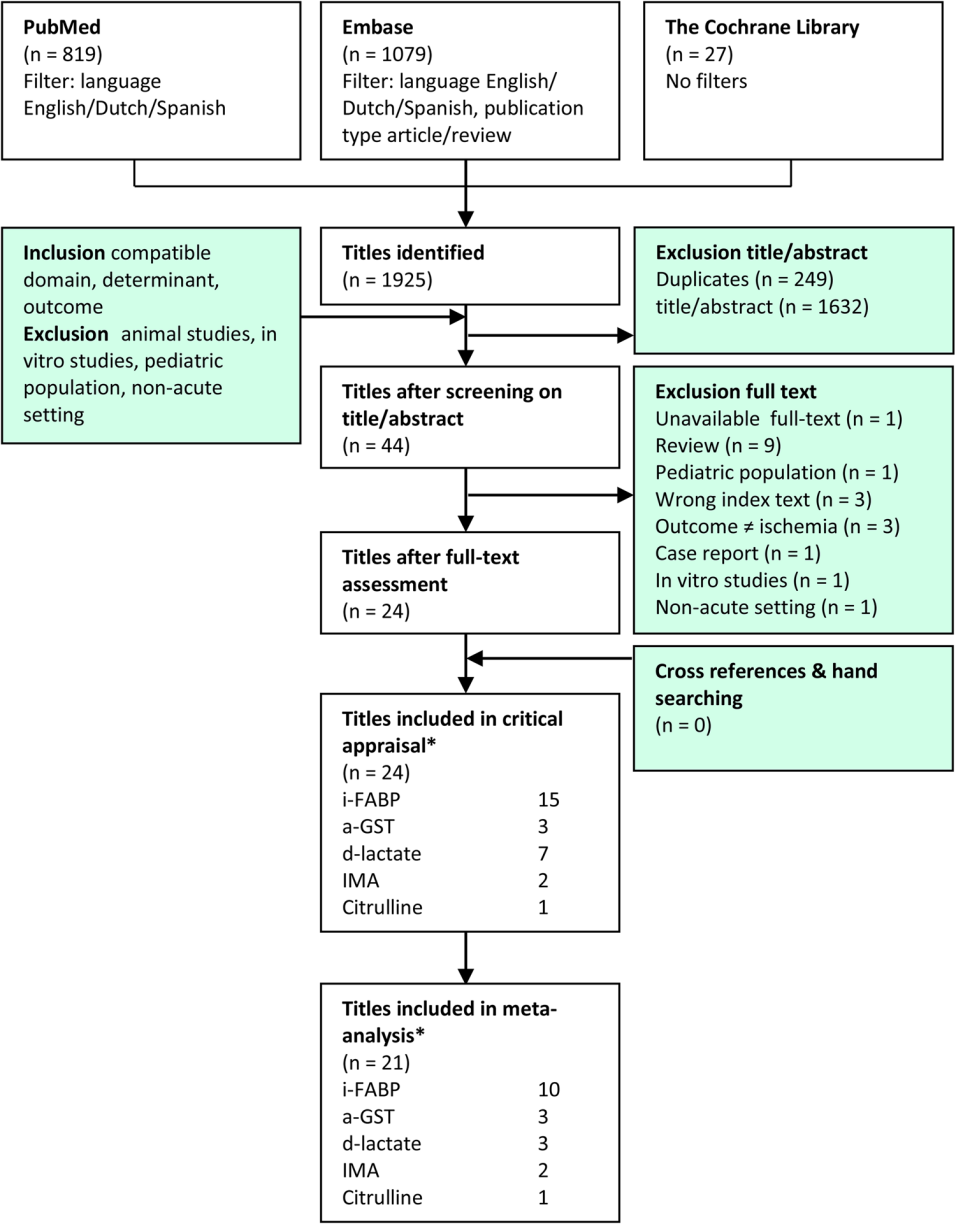
Search strategy and flow chart. Some authors investigated multiple biomarkers

A systematic search in Embase, PubMed, and the Cochrane Library was performed to identify all relevant literature published before November 2016 (Supplementary Appendix 1). Only studies written in English, Dutch, French, Spanish, or German were included. Duplicates were removed using Covidence^®^ software (Melbourne, Australia, 2015) [27]. Two reviewers (NT, AP) screened potential relevant articles based on title and abstract, and according to pre-defined inclusion and exclusion criteria (Fig. 1). Cross-references of relevant reviews were screened.

### Selection criteria

Eligible studies were observational or case-controlled studies that assessed the diagnostic accuracy of the investigated serological biomarkers in patients with AMI suspected on clinical grounds. AMI was ideally confirmed by laparotomy, colonoscopy, or autopsy. A study was included in the meta-analysis if true positive, false positive, true negative, and false negative test results could be derived to pool and calculate diagnostic accuracy standards directly from published data.

### Assessment of methodological quality

Using modified criteria based on the QUADAS-2 tool and the Cochrane checklist for diagnostic studies, two authors (NT, AP) independently critically appraised the selected articles for risk of bias (validity) and applicability [27–30]. Judgments were discussed after which consensus was reached. Risk of bias was considered high in case of a low score on ≥2 items, moderate in case of a low score in 1–2 items, and low when all items were scored moderate or high. Verification bias was considered of limited importance, as AMI will eventually be either diagnosed by laparotomy or autopsy. In case of full clinical recovery without invasive intervention, it was safely assumed that no mesenteric infarction of clinical importance was present. Applicability was considered low in case of absent extractable data or poor representation of domain.

### Data synthesis and statistical analysis

Data from individual studies and pooled results are expressed as means with 95% confidence intervals (CI). Data to construct two-by-two contingency tables were retrieved to calculate diagnostic accuracy standards. Meta-DiSc^®^ version 1.4 (Meta-DiSc Software, Madrid, Spain) [31] was used to calculate pooled sensitivity and specificity, and positive- and negative-likelihood ratios (LR). A random-effect model according to DerSimonian and Laird was used for meta-analysis [32]. When a two-by-two table included a zero cell, 0.5 was added [31, 33].

Data derived by meta-analyses are presented as forest plots. Forest plots display the diagnostic probabilities of individual studies and the corresponding 95% CI. Units for d-lactate were converted from mcg/mL to mmol/L using 90 g/mol as the molar mass for lactate. Study heterogeneity was determined by the *χ* ^2^ tests and *I* ^2^ measures. Studies with an *I* ^2^ value below 25% were considered homogeneous, 26–50 and 51–75% as low and moderate and over 75% as high heterogeneity, respectively [34]. A *p* value of <0.05 was considered statistically significant. Results were reported in accordance with the PRISMA recommendations [35]. The protocol for this systematic review and meta-analysis was registered on PROSPERO (CRD42016052163) [36].

## Results

### Search and selection criteria

The study includes results of electronic searches up to November 2016. Figure 1 depicts the selection of articles included in the analysis. A total of 1925 papers were identified of which 44 were retrieved for full-text review. A total of 15 papers on I-FABP, seven on d-lactate, three on α-GST, two on IMA, and one on citrulline were ultimately selected for final critical appraisal. In one article [37], I-FABP, d-lactate, and α-GST were studied simultaneously. In two papers, both I-FABP and d-lactate were studied [38, 39].

### Critical appraisal

Results of critical appraisal are shown in Table 1. After critical appraisal, three papers were excluded from the final analysis [49, 50, 52]. Camkiran studied plasma I-FABP levels in 35 patients undergoing elective coronary artery bypass; however, none of the patients developed AMI. The study by Lieberman was excluded due to low applicability and high risk of bias. As for Collange, no AMI was observed in patients undergoing elective infrarenal aortic aneurysm surgery.

**Table 1:** Critical appraisal

	References	Study design	Patient selection	Threshold	Blinded index test results	Valid reference test	Disease progression	Verification	Withdrawal	Risk of bias	Representative patient sample	Extractable data	Applicability
I-FABP	Block et al. [37]	Cohort	●	○	●	●	●	○	●	Low	●	◐	Moderate
	Cronk et al. [40]	Cohort	●	◐	●	●	●	◐	●	Low	●	●	High
	Güzel et al. [41]	Case–control	◐	●	●	●	○	●	●	Moderate	●	●	High
	Kanda et al. [15]^a^	Case–control	◐	●	●	●	○	○	●	Moderate	●	●	High
	Kanda et al. [42]	Cohort	●	●	●	●	○	◐	●	Low	●	●	High
	Kittaka et al. [43]	Cohort	●	●	●	●	●	◐	●	Low	●	●	High
	Matsumoto et al. [44]	Cohort	●	●	●	●	●	◐	●	Low	●	●	High
	Matsumoto et al. [45]	Cohort	●	●	●	●	○	●	●	Low	●	●	High
	Shi et al. [38]	Cohort	●	●	●	●	●	◐	●	Low	●	●	High
	Thuijls et al. [46]	Cohort	●	●	●	●	●	◐	●	Low	●	●	High
	Uzun et al. [47]	Case–control	◐	●	●	◐	○	○	●	High	●	●	High
	Vermeulen et al. [48]	Cohort	●	●	●	●	●	◐	●	Low	●	●	High
	Voort et al. [39]^a^	Cohort	●	○	●	●	●	◐	●	Low	●	◐	Moderate
	Camkiran et al. [49]	Cohort	●	○	●	○	●	○	●	High	●	○	Low
	Lieberman et al. [50]^a^	Case–control	◐	○	●	○	○	○	●	High	◐	◐	Low
d-Lactate	Assadian et al. [51]	Cohort	●	○	●	●	●	●	●	Low	●	◐	High
	Block et al. [37]	Cohort	●	◐	●	●	●	◐	●	Low	●	●	High
	Collange et al. [52]	Cohort	●	◐	●	●	●	●	●	Low	●	○	Low
	Murray et al. [53]	Case–control	◐	◐	○	●	○	●	●	Moderate	●	●	High
	Poeze et al. [21]	Case–control	◐	◐	●	◐	○	◐	●	High	●	●	High
	Shi et al. [38]	Cohort	●	●	●	●	●	●	●	Low	●	●	High
	Voort et al. [39]^a^	Cohort	●	○	●	●	●	◐	●	Low	●	◐	Moderate
α-GST	Block et al. [37]	Cohort	●	◐	●	●	●	◐	●	Low	●	●	High
	Delaney et al. [54]	Case–control	◐	●	●	●	●	●	●	Low	●	●	High
	Gearhart et al. [55]	Cohort	●	◐	●	●	●	◐	◐	Low	●	●	High
IMA	Gunduz et al. [56]	Case–control	◐	●	●	●	●	●	●	Low	●	●	High
	Polk et al. [57]	Cohort	●	●	●	●	●	●	●	Low	●	●	High
C	Kulu et al. [58]	Case–control	○	●	●	●	○	●	●	Low	●	●	High

Patient selection: ● consecutive order, well described in- and exclusion criteria ◐ case–control with consecutive case selection ○ inappropriate exclusions. Threshold: ● based on ROC-analysis ◐ pre-specified ○ not reported. |Blinded index test results: ● yes ○ no/not reported. Valid reference standard: ● surgery, endoscopy, autopsy, full clinical recovery ◐ CT scanning, lab findings ○ none/not reported. Disease progression: ● < 12 h ○ ≥ 12 h/not reported. Verification: ●all patients received both index and reference test. Reference test was the same for all patients ◐ selected patients received equal reference tests ○ selected patients received different reference tests. Withdrawal: ● no loss to follow up ◐loss to follow up, reasons given ○ loss to follow up without reasons given/not reported. Representative patient sample: ● patients with suspected AMI ◐ healthy control group ○ non-matching domain. Extractable data: ● 2 × 2 table data extractable ◐ levels of biomarkers reported, no 2 × 2 data extractable ○ only correlation, no data on AMI

^a^Articles found by hand searching

### Clinical results

Table 2 presents the characteristics of the included studies. The final analysis includes 21 studies evaluating 1670 patients for AMI. The pooled prevalence is 22.0%. The pre-test probability of AMI varied (4.1–53.7%) between studies, reflecting variations in domains. In 15 studies, patients presenting with an acute abdomen were studied (*n* = 1436, mean prevalence 21.4%). Two papers [40, 43] evaluated patients with bowel obstruction (*n* = 58, 41.4%). An additional four papers [21, 39, 48, 51] included patients at risk for NOMI. Table 3 presents the accuracy data extracted from each individual study. Table 4 presents pooled sensitivity and specificity for each biomarker.

**Table 2:** Characteristics of included studies

(A) Study	Country	No. of patients	Study population	Timing of blood sampling	Reference test	I-FABP measurement	Prevalence AMI (%)
I-FABP studies							
Block et al. [37]	Sweden	71	Acute abdomen	At presentation	Laparotomy, histopathology, autopsy, clinical evaluation, radiological findings	ELISA (Hycult Biotechnology b.c., Uden, The Netherlands)	14.1
Cronk et al. [40]	USA	21	Mechanical bowel obstruction	At presentation	Laparotomy	ELISA (Hycult Biotechnology b.c., Uden, The Netherlands)	14.3
Güzel et al. [41]	Turkey	57	Acute abdomen	NR	Laparotomy and histopathology	ELISA (Hycult Biotechnology b.c., Uden, The Netherlands)	47.4
Kanda et al. [42]	Japan	361	Acute abdomen	Within 24 h after presentation	Laparotomy	ELISA, rabbit and mice anti-human I-FABP polyclonal antibodies	14.4
Kittaka et al. [43]	Japan	37	Small bowel obstruction	At presentation	Laparotomy	ELISA, rabbit and mice anti-human I-FABP polyclonal antibodies	45.9
Matsumoto et al. [44]	Japan	146	Acute abdomen	Directly after initial assessment	Laparotomy, autopsy, clinical evaluation	Recombinant I-FABP assay (Sumitomo Pharma Biomedical Centre, Osaka, Japan)	16.4
Matsumoto et al. [45]	Japan	48	Pneumatosis intestinalis	At presentation	Laparotomy	Osaka	39.0
Shi et al. [38]	China	272	Acute abdomen	At presentation	Laparotomy, autopsy, CT scanning, colonoscopy	Standard ELISA kits NOS	14.3
Thuijls et al. [46]	The Netherlands	50	Acute abdomen	At presentation	Laparotomy/autopsy with PA, consensus	ELISA (Hycult Biotechnology b.c., Uden, The Netherlands)	47.8
Uzun et al. [47]	Turkey	171	Acute abdomen	At presentation	NR	ELISA (Hycult Biotechnology b.c., Uden, The Netherlands)	4.1
Vermeulen Windsant et al. [48]	The Netherlands	96	Major aortic surgery	At 7 time points peri-operatively	Laparotomy	ELISA (Hycult Biotechnology b.c., Uden, The Netherlands)	4.2
Kanda et al. [15]	Japan	61	Acute abdomen	At presentation	Laparotomy	ELISA (Niigata University School of Medicine, Niigata, Japan)	21.3
van der Voort et al. [39]	The Netherlands	44	ICU patients	When AMI was considered in the diagnostic work up	Laparotomy, histopathology, endoscopy, CT scan	ELISA (Hycult Biotechnology b.c., Uden, The Netherlands)	52

(B) Study	Country	No. of patients	Study population	Timing of blood sampling	Reference test	Biomarker measurement	Prevalence AMI (%)
d-Lactate studies							
Block et al. [37]	Sweden	71	Acute abdomen	At presentation	Laparotomy, histopathology, autopsy, clinical evaluation, radiological findings	Spectrophotometry using R-BIOPHARM AG, Darmstadt, Germany)	14.1
Assadian et al. [51]	Austria	12	Open aortic reconstruction	At 4 time points peri-operatively	Histopathology (biopsy during sigmoidoscopy)	Enzymatic reactions using d-Lactate dehydrogenase and alanine aminotransferase	25
Shi et al. [38]	China	272	Acute abdomen	At presentation	Laparotomy, autopsy, CT scanning, colonoscopy	Standard ELISA kits	14.3
Murray et al. [53]	USA	31	Acute abdomen scheduled for surgery	Preoperative	Laparotomy	Spectrophotometrically	29.0
Poeze et al. [21]	The Netherlands	24	Major emergency aortic surgery	Postoperatively at admission ICU	Colonoscopy	Enzymatic reactions using d-Lactate dehydrogenase and alanine aminotransferase	45.8
van der Voort et al. [39]	The Netherlands	44	ICU patients	When AMI was considered in the diagnostic work up	Laparotomy, histopathology, endoscopy, CT scan	Spectrophotometrically	52
α-GST studies							
Block et al. [37]	Sweden	71	Acute abdomen	At presentation	Laparotomy, histopathology, autopsy, clinical evaluation, radiological findings	ELISA IHEPKIT, Biotrin International, Dublin, Ireland)	14.1
Gearhart et al. [55]	USA	54	Patients with clinical suspicion for AMI	At presentation	Colonoscopy, angiography, laparotomy, autopsy	ELISA IHEPKIT, Biotrin International, Dublin, Ireland)	53.7
Delaney et al. [54]	Ireland	26	Acute abdomen	At presentation	Autopsy, laparotomy, other definitive investigation, return to full health	ELISA IHEPKIT, Biotrin International, Dublin, Ireland)	46.2
IMA studies							
Gunduz et al. [56]	Turkey	14	Thromboembolic occlusion SMA	On admission	Laparotomy	Cobalt–Albumin-binding assay (zie references #10)	50.0
Polk et al. [57]	Sweden	26	Possible AMI scheduled for laparotomy	Within 1 h preoperatively	Laparotomy	Cobalt–Albumin-binding assay (zie references #3)	46.2
Citrulline studies							
Kulu et al. [58]	Turkey	48	Acute abdomen	At presentation	Laparotomy	Amino Acids LC–MS/MS analysis Kit, Zivak Technologies, Turkey	47.9

*NR* not reported, *ICU* intensive care unit, *NOS* not otherwise specified, *AMI* acute mesenteric ischemia

**Table 3:** Data analysis

	Mean control^a^	Mean AMI^b^	*p* value	Cut-off level	TP	FP	TN	FN	PPV^c^	NPV^d^
I-FABP Uden kit (ng/mL)										
Block et al. [37]	0.050 (0.0–0.197)	0.186 (0.0–0.613)	0.58	N/A	N/A	N/A	N/A	N/A	N/A	N/A
Cronk et al. [40]	0.281	1.772	N/A	0.1	3	4	14	0	0.43 (0.10–0.82)	1.00 (0.77–1.00)
Güzel et al. [41]	0.08 (0.01–0.20)	0.421 (0.040–5.0)	<0.001	0.09	24	0	30	3	1.00 (0.86–1.00)	0.09 (0.02–0.24)
Thuijls et al. [46]^f^	0.109 [0.04–1.691]	0.653 [0.04–74.711]	0.02	0.268	15	7	17	7	0.68 (0.45–0.86)	0.71 (0.49–0.87)
Uzun et al. [47]	0.170 ± 0.543	0.709 ± 0.669	N/A	0.145	5	9	155	2	0.36 (0.13–0.65)	0.99 (0.95–1.00)
Vermeulen Windsant et al. [48]	N/A	N/A	N/A	0.815	4	0	92	0	1.00 (0.40–1.00)	1.00 (0.96–1.00)
van der Voort et al. [39]^f^	1.020	2.872	0.98	N/A	N/A	N/A	N/A	N/A	N/A	N/A
I-FABP Osaka kit (ng/mL)										
Kanda et al. [15]	25.1 ± 3.6	265.8 ± 111.3	<0.0	100	7	0	48	6	1.00 (0.59–1.00)	0.89 (0.77–0.96)
Kanda et al. [42]	5.8 ± 15.6	40.7 ± 117.9	<0.0001	3.1	41	81	228	11	0.34 (0.25–0.43)	0.95 (0.92–0.98)
Kittaka et al. [43]	1.6	18.5	<0.001	6.5	15	1	15	6	0.94 (0.70–1.00)	0.71 (0.48–0.89)
Matsumoto et al. [44]	2.5 (0.2–56.7)	31.0 (1.1–498.4)	<0.01	9.1	20	13	109	4	0.61 (0.42–0.77)	0.96 (0.91–0.99)
Matsumoto et al. [45]	3.2 [1.7–6.7]	15.5 [5.3–52.9]	<0.001	9.7	19	6	37	8	0.76 (0.59 – 0.87)	0.82 (0.72 – 0.89)
Shi et al. [38]	33.9 ± 12.6	113.8 ± 46.3	<0.001	93.07	30	59	174	9	0.34 (0.24–0.45)	0.95 (0.91–0.98)
d-Lactate (mmol/L)										
Block et al. [37]	0.03 (0.02–0.07)	0.05 (0.03–0.10)	0.20	0.20	9	47	14	1	0.16 (0.08–0.28)	0.93 (0.68–1.00)
Shi et al. [38]^f^	0.15 ± 0.06	0.66 ± 0.29	<0.001	0.38	26	33	200	13	0.44 (0.31–0.58)	0.94 (0.90–0.97)
van der Voort et al. [39]^e^	0.65 [0.37–0.94]	0.79 (0.49–1.16)	0.003^g^	N/A	N/A	N/A	N/A	N/A	N/A	N/A
	0.41 [0.11–0.75]	0.56 (0.27–0.77)	0.46^h^							
Murray et al. [53]^f^	0.12 ± 0.04	0.36 ± 0.04	<0.0005	0.22	8	1	19	3	0.89 (0.52–1.00)	0.86 (0.65–0.97)
Assadian et al. [51]	1.25 ± 0.61	3.03 ± 1.65	0.035	N/A	N/A	N/A	N/A	N/A	N/A	N/A
Poeze et al. [21]	0.21 ± 0.06	0.32 ± 1.0	<0.01	0.2	9	3	10	2	0.75 (0.43–0.95)	0.83 (0.52–0.98)
α-GST (ng/ml)										
Block et al. [37]	1.3 (1.1–2.8)	1.7 (0.7–4.2)	0.21	4	2	9	53	8	0.18 (0.02–0.52)	0.87 (0.76–0.94)
Gearhart et al. [55]	2.2 (1.0–3.0)	22.2 (7.0–126.0)	0.001	4	25	4	15	10	0.86 (0.68–0.96)	0.60 (0.39–0.79)
Delaney et al. [54]	1.6 (0.8–2.2)	75.8 (22.4–153.0)	<0.0001	4	12	2	12	0	0.86 (0.57–0.98)	1.00 (0.74–1.00)
IMA (ABSU)										
Polk et al. [57]	0.31 ± 0.02	0.52 ± 0.04	<0.0002	0.35	12	2	12	0	0.86 (0.57–0.98)	1.00 (0.74–1.00)
Gunduz et al. [56]	0.163 ± 0.025	0.264 ± 0.057	0.003	0.188	6	1	7	0	0.86 (0.42–1.00)	1.00 (0.59–1.00)
Citrulline (nmol/ml)										
Kulu et al. [58]	32.8 ± 3.0	21.7 ± 3.1	0.01	15.8	9	0	25	14	1	0.64 (0.56–0.71)

*N/A* not applicable, *TP* true positive, *TN* true negative, *FP* false positive, *FN* false negative, *ABSU* absorbance units

^a^Numbers between brackets represent 95% confidence intervals. Means are presented with standard deviation

^b^Acute mesenteric ischemia

^c^Positive predictive value

^d^Negative predictive value

^e^Median [IQR] are presented

^f^Values were converted to mmol/L by multiplying by 0.0111

^g^Ischemia vs. non-ischemia

^h^Ischemia-likely vs. ischemia-unlikely

**Table 4:** Meta-analysis

	No. of studies	Sensitivity	*I* ^2^ (%)	*p* value	Specificity	*I* ^2^ (%)	*p* value	Positive LR^a^	*I* ^2^ (%)	*p* value	Negative LR	*I* ^2^ (%)	*p* value
I-FABP (Uden kit)	4	0.790 (0.665–0.885)	16	0.312	0.913 (0.870–0.946)	82	0.001	6.368 (2.100–18.534)	79	0.003	0.262 (0.130–0.543)	41	0.146
I-FABP (Osaka kit)	6	0.750 (0.679–0.812)	0	0.463	0.792 (0.762–0.820)	87	0.000	4.577 (2.910–7.197)	75	0.001	0.321 (0.249–0.413)	0	0.629
d-lactate	3	0.717 (0.586–0.825)	20	0.288	0.742 (0.690–0.790)	98	0.000	3.621 (0.770–17.035)	97	0.000	0.371 (0.249–0.552)	0	0.845
α-GST	3	0.678 (0.542–0.795)	88	0.000	0.842 (0.753–0.909)	0	0.792	3.27 (1.50–7.16)	27	0.252	0.40 (0.11–1.49)	90	0.000
IMA	2	0.947 (0.740–0.999)	0	0.739	0.864 (0.651–0.971)	0	0.906	6.931 (2.37–24.24)	0	0.935	0.064 (0.02–0.48)	0	0.742

Numbers between brackets represent 95% confidence intervals

*I* ^*2*^ inconsistency (*I*-square)

^a^Likelihood ratio

### I-FABP

There are 13 studies including 1435 patients that examine the performance of I-FABP for the diagnosis of intestinal ischemia. The overall prevalence is 18.3%. Laparotomy (or autopsy) was performed in 1099 patients, including all patients with AMI. Plasma I-FABP was measured using two different kits. Since cut-off values differed greatly between these groups, data were pooled per kit.

**Fig. 2:**
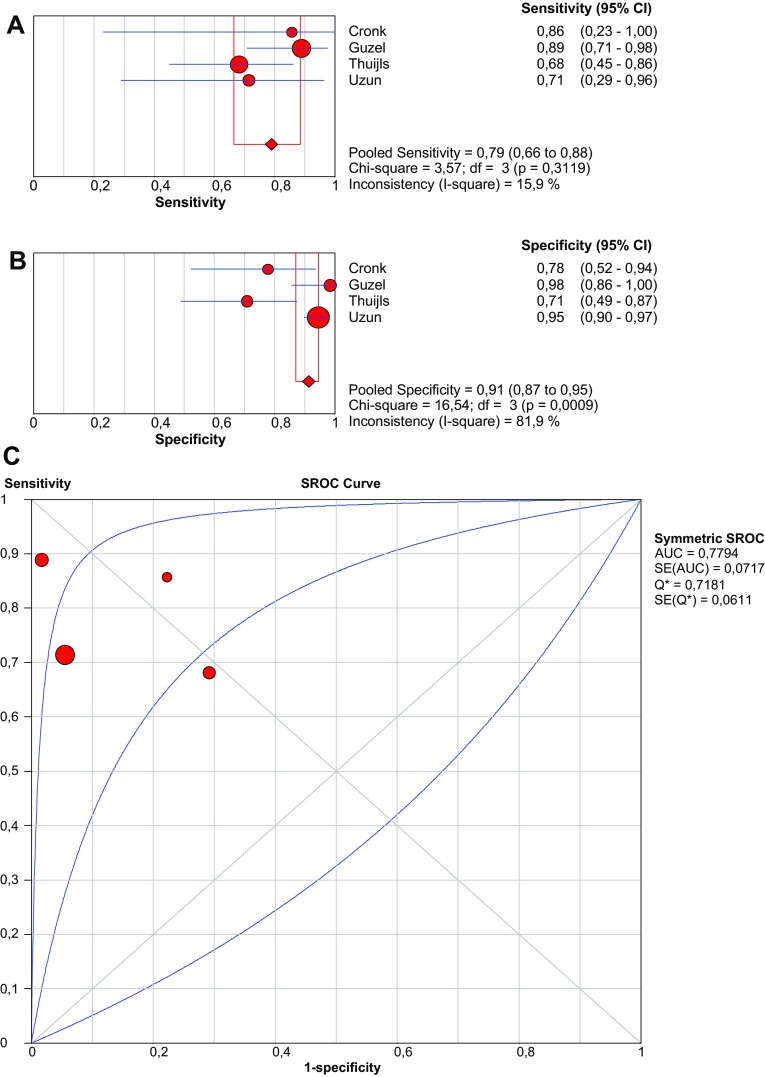
Forest plots and SROC curve of I-FABP (Uden kit) to detect acute mesenteric ischemia. *SROC* summary receiver-operating characteristic, *AUC* area under curve, *SE* sensitivity

In seven studies, a human ELISA kit (HyCult Biotechnologie, Uden, The Netherlands) was used. The cut-off values of these studies vary between 0.09 and 0.815 ng/mL. The studies by Block [I-FABP difference non-significant between patients with and without AMI (*p* = 0.58)] and Van der Voort [I-FABP with AMI: 2.872 ng/mL (95% CI 0.229–4.340) vs. I-FABP without AMI: 1.020 ng/mL (95% CI 0.239–5.324), *p* = 0.98] were not included as calculation of diagnostic accuracy standards was not possible. In four studies that examined the accuracy of I-FABP in patients presenting with acute abdomen, two-by-two contingency tables could be derived [40, 41, 46, 47]. Pooled sensitivity and specificity are 79.0% (95% CI 66.5–88.5) and 91.3% (95% CI 87.0–94.6), respectively (Fig. 2). Vermeulen et al. studied patients after thoracic, thoracoabdominal or abdominal aneurysm repair. They find a sensitivity and specificity of both 100%, with a cut-off value of 0.815 ng/ml.

**Fig. 3:**
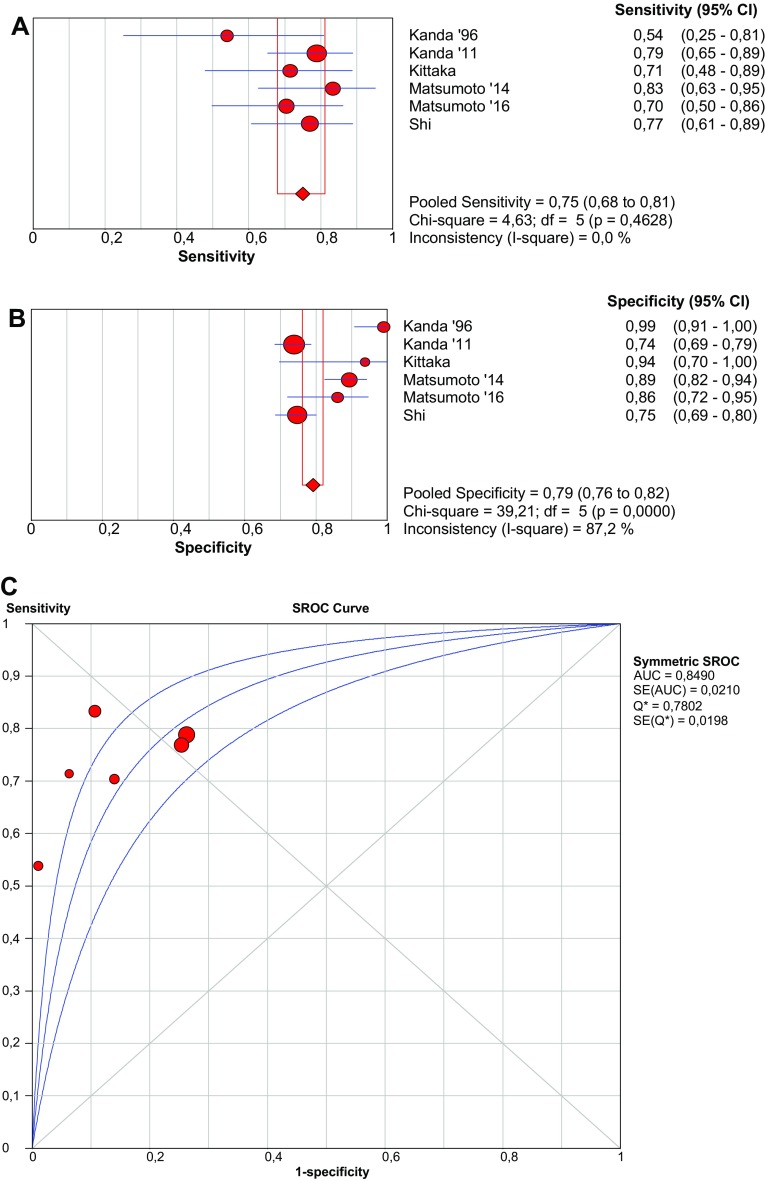
Forest plots and SROC curve of I-FABP (Osaka kit) to detect acute mesenteric ischemia. *SROC* summary receiver-operating characteristic, *AUC* area under curve, *SE* sensitivity

In six studies, plasma I-FABP levels were measured using a sandwich ELISA system with rabbit anti-human I-FABP polyclonal antibodies in the solid phase and mouse anti-human I-FABP monoclonal antibodies in the liquid phase (D.S. Pharma Biomedical Co., Ltd., Osaka, Japan). Although in one study [38], the exact ELISA test used was unclear; the reference values were comparable to the studies in which the Osaka kit was used. Therefore, we combined results from this study with the other Osaka kit studies. Pooled sensitivity and specificity are 75.0% (95% CI 67.9–81.2%) and 79.2% (95% CI 76.2–82.0), respectively (Fig. 3). The cut-off value varies from 3.1 and 100 ng/mL.

### d-Lactate

**Fig. 4:**
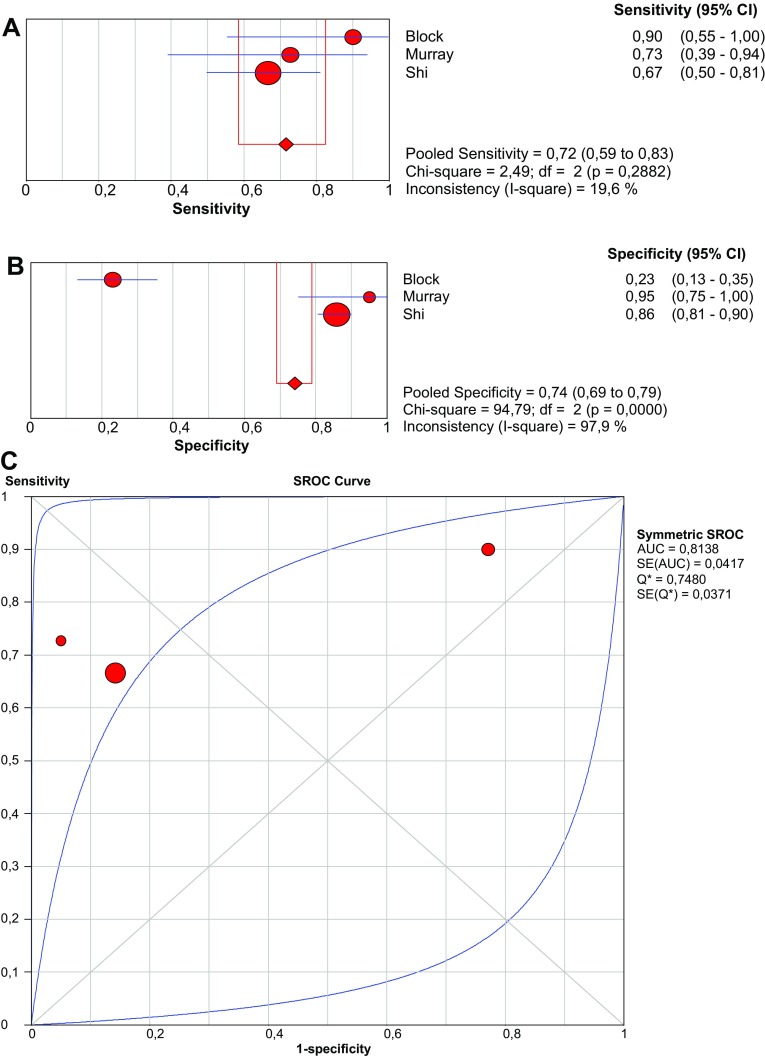
Forest plots and SROC curve of d-lactate to detect acute mesenteric ischemia. *SROC* summary receiver-operating characteristic, *AUC* area under curve, *SE* sensitivity

Six studies focused on d-lactate as a serological biomarker for AMI. Pooled prevalence of AMI is 17.3%. Three studies examined patients with an acute abdomen [37, 38, 53]. Pooled sensitivity and specificity are 71.7% (95% CI 58.6–82.5%) and 74.2% (95% CI 69.0–79.0%), respectively (Fig. 4).

In addition, three authors investigated patients at risk for NOMI. Poeze and colleagues studied the accuracy of d-lactate in patients after repair of ruptured abdominal aortic aneurysm (AAA), and find a sensitivity and specificity of 82 and 77%, respectively. Assadian et al. studied the presence of AMI after repair of ruptured or symptomatic AAA. A significant difference in serum d-lactate is found at 2, 24 and 48 h postoperatively (*p* = 0.045, *p* = 0.027 and *p* = 0.035, respectively). Van der Voort et al. calculated mean d-lactate levels in critically ill ICU patients suspected for AMI. A significant difference is found in d-lactate levels between patients with proven and likely AMI versus unlikely and non-ischemic patients (*p* = 0.003).

### α-GST

**Fig. 5:**
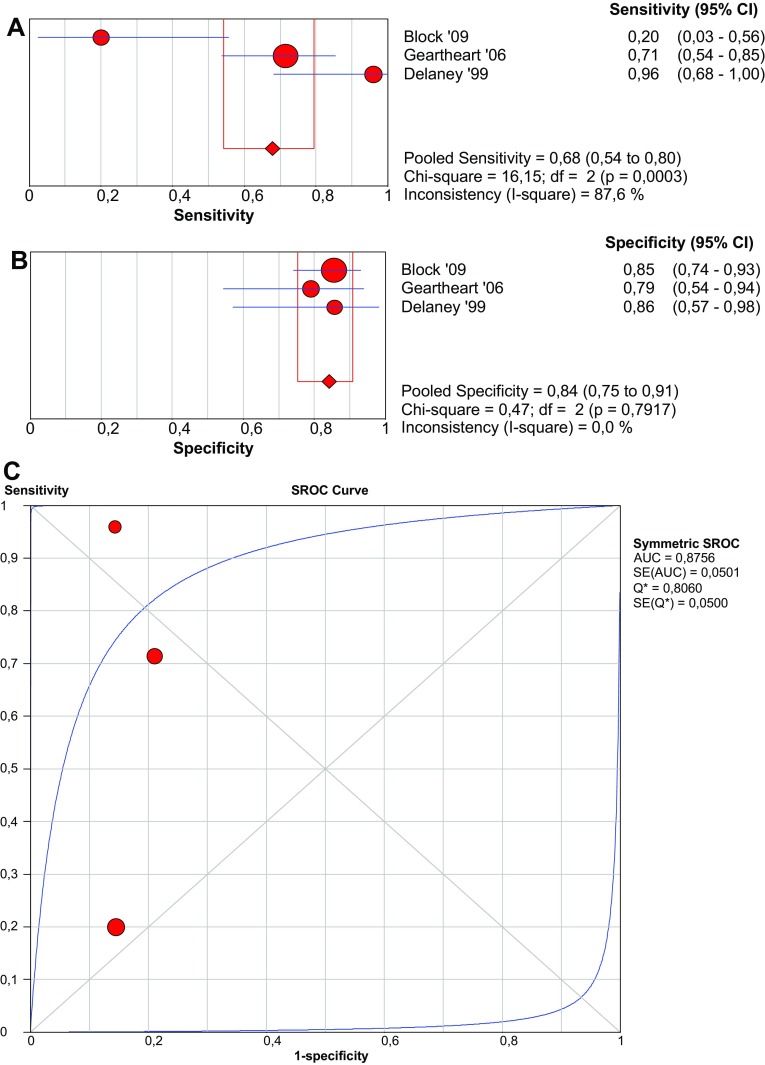
Forest plots and SROC curve of alpha-GST to detect acute mesenteric ischemia. *SROC* summary receiver-operating characteristic, *AUC* area under curve, *SE* sensitivity

Three studies, including 151 patients with suspected AMI, addressed the performance of α-GST for the diagnosis of AMI. The cut-off value of α-GST was pre-defined as 4 ng/mL in all studies. Pooled sensitivity and specificity are 67.8 (95% CI 54.2–79.5%) and 84.2% (95% CI 75.3–90.9%), respectively (Fig. 5).

### IMA

**Fig. 6:**
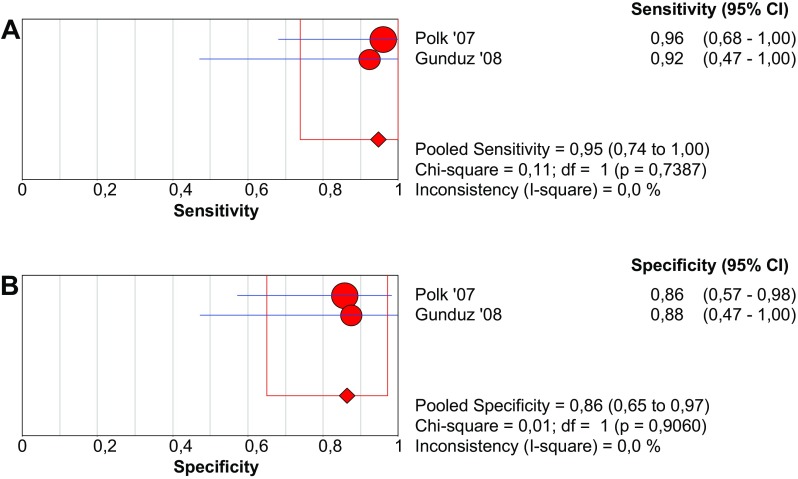
Forest plots and SROC curve of IMA to detect acute mesenteric ischemia

Gunduz et al. determined whether IMA is elevated in patients with AMI. In their case-controlled study of seven cases with thromboembolic occlusion of the superior mesenteric artery, they find a statistically different concentration of IMA compared to seven controls (*p* = 0.003). The cut-off value of 0.188 ABSU yields a positive predictive value (PPV) and negative predictive value (NPV) of 0.86 (95% CI 0.42–1.00) and 1.00 (95% CI 0.59–1.00). Polk et al. studied the value of IMA in patients presenting with an acute abdomen and calculate a PPV and NPV of 0.86 (95% CI 0.57–0.98) and 1.00 (0.74–1.00), respectively. Pooled sensitivity and specificity are 94.7 (95% CI 74.0–99.9%) and 86.4% (95% CI 65.1–97.1%), respectively (Fig. 6).

### Citrulline

Kulu et al. investigated the diagnostic accuracy of citrulline for AMI in patients with acute abdomen. Specificity and sensitivity of 100 and 39% are found, respectively.

## Discussion

Usage of serological markers as screening tools, either to contribute to the present diagnostic armamentarium, or to replace presently used diagnostic tests, should depend on the clinical setting and the pre-test probability. The incidence of AMI in patients presenting with acute abdominal pain at the emergency department is relatively low compared to patients in the ICU, and differential diagnosis is comprehensive. The CT (angiography) scan can be a valuable diagnostic tool in diagnosing occlusive AMI [59]. In NOMI, however, radiological findings are often less specific [60]. In addition, CT scanning can be contraindicated in patients with impaired kidney function or contrast allergy. Especially, in these patients, a screening serum test would be helpful. In this systematic review and meta-analysis, six serological biomarkers were analysed for their capability to diagnose AMI: I-FABP, d-lactate, α-GST and IMA, and citrulline. Citrulline (100%), I-FABP (Uden kit, 91%), and IMA (86%) demonstrate high specificity, suggesting that when the levels are below the defined cutoff, chances of AMI are low. However, false negative rates of 9–14% in I-FABP and IMA are still debatable considering the consequences of delaying laparotomy and the impact on the final outcome.

Compared to a meta-analysis performed by Evennett et al., we separated the different kits in our analyses and added eight new studies in the evaluation of I-FABP. In the follow-up of patients who were diagnosed with AMI and in whom a segment of questionable viable intestine was not resected, interval I-FABP levels can support the decision to perform a second-look operation [40]. Therefore, interval postoperative I-FABP measurement may be useful. Although the true incidence of clinically relevant AMI in patients presenting to the emergency department with acute abdominal pain is unknown [38], Thuijls et al. studied a population with a relatively high pre-test probability of AMI of 47.8%, compared to the other studies. This might have led to an overestimation of the predictive contribution of I-FABP in the diagnosis of AMI in these patients. A poor renal function delays the clearance of plasma I-FABP [43]. Except for Vermeulen, none of the selected studies excluded this group nor described renal function in baseline tables on patient characteristics. This may have led to information bias.

In d-lactate, summary sensitivity and specificity are relatively low with 71.7 (95% CI 58.6–82.5) and 74.2 (95% CI 69.0–79.0), respectively. Previously, a meta-analysis by Evennett [33] reports a sensitivity and specificity of 82 and 48%, respectively. The studies on d-lactate scored low on heterogeneity, because only studies with patients with an acute abdomen as a domain were pooled. Except for the results from Shi, none of the other studies calculate an optimal threshold according to the results. Therefore, the pooled sensitivity and specificity do not represent the most optimal values.

Although results are fairly promising (sensitivity 67.8%, specificity 84.2%), α-GST may be non-specific for AMI, as it may also be released by the liver during oxidative stress [33]. Plasma levels of α-GST may increase in patients with shock, acute, or chronic liver failure and hepatitis. These factors may influence the diagnostic accuracy in these specific patient groups, however, which have not yet been studied extensively. Since α-GST is especially specific for small bowel ischemia, isolated colonic ischemia may go underdiagnosed. This may explain the relatively low pooled sensitivity. Therefore, it seems attractive to combine α-GST with a marker more specific for the colon. Moreover, the pre-test prevalence of AMI was relatively high in two out of three studies on α-GST, leading to a limited external validity for patients with lower pre-test probabilities.

IMA demonstrated the highest sensitivity (94.7%). Nevertheless, the patient groups were small and pre-test probability was high (48.7%), because patients with a known thromboembolic occlusion were included as well. These factors may have led to an overestimation of the diagnostic accuracy. IMA levels may also be elevated in patients with cardiac ischemia. In patients in the ICU, cardiac ischemia may be present due to secondary ischemia caused by severe illness. In future research, it should be acknowledged that this might convey risk of confounding.

Only one study was found on citrulline. High specificity (100%) and positive predictive value are reported. These results should be interpreted with caution, since there was a high pre-test probability. Nevertheless, citrulline remains a potential accurate marker for AMI, since it has been shown to be a reliable marker of functional enterocyte mass [24], prognostic value of mortality in the ICU [60], and NOMI after cardiac arrest [61].

### General strengths and limitations

Strengths of our review are the extensive search and critical review by independent authors. Moreover, cross-references of relevant reviews were checked to include all relevant articles. All studies on I-FABP have a low risk of interval bias because of a narrow interval between the diagnosis of AMI and the obtainment of blood samples for the determination of I-FABP levels. In addition, there is a low risk of review bias in studies on I-FABP, α-GST, and IMA, since all included studies blindly assessed the index test. Therefore, the decision whether or not to perform a laparotomy was not influenced by test results. Murray did not report blind assessment of index test results. AMI has multiple aetiologies. Therefore, in the analysis, data were pooled separately according to aetiology.

Several limitations should be mentioned. In the included studies not all patients underwent a laparotomy, which is regarded as the gold standard for AMI. Instead, diagnosis was based on combinations of clinical features, CT findings, colonoscopy, and regular laboratory findings. Partial verification bias may have been introduced. However, the effect will be limited, as clinically relevant AMI typically needs surgical intervention, or will result in a poor outcome that is detectable in the studies. Furthermore, this systematic review is limited by inter-study variation in cut-off values. An overall cut-off value could not be given for all biomarkers, except for α-GST. The serum values of the biomarkers are influenced by several factors. First, the severity of intestinal damage may result in more divergent plasma levels. The timing of sampling after the onset of symptoms varied among studies, potentially leading to interval bias. In addition, the previous colonic surgery or chronic kidney failure may affect the base-level and clearance of the investigated biomarkers. For example, in short bowel syndrome, the citrulline levels are generally lower than in the general population [62], which may bias test results. In addition, high levels of citrulline now reflect plasma clearance, and may overestimate functional enterocyte mass. In addition, the method of measurement may be of influence as well. For example, different ELISA kits were used in the studies on I-FABP. To circumvent the effect of bias caused by variation in I-FABP measurements, we considered the Uden and Osaka kits as different diagnostic tests and performed meta-analysis only on the separate groups. Variation within the same kits may be caused by inappropriate storage of samples, incorrect analyses, and inter-laboratory variation. However, no indications of variation within the same kits were found in the description of methods of these studies. Another limitation of this review is that studies with a small study population are also included. This may have incorrectly influenced the pooled diagnostic accuracy standards, leading to an over- or underestimation of results.

In conclusion, this systematic review and meta-analysis presents pooled estimates of I-FABP, d-lactate, α-GST, and IMA as serological biomarkers for the diagnosis of acute mesenteric ischemia. The best pooled performance is demonstrated for IMA and I-FABP (Uden kit). Citrulline is a promising marker as well with high reported specificity. Results should be interpreted with caution due to the heterogeneous and small patient populations studied. As both positive and negative predictive values do not demonstrate optimal performance, it is too early to consider them to replace other diagnostic modalities such as CT angiography. Possibly, combination of multiple biomarkers may lead to a synergistic diagnostic performance. Diagnostic models including both clinical, radiological, and laboratory tests may eventually facilitate identification of those patients with AMI who need urgent surgical treatment potentially reducing morbidity and mortality from this life-threatening disease.

## Electronic supplementary material

Below is the link to the electronic supplementary material.

Supplementary material 1 (DOCX 120 kb)

## Abbreviations

I-FABP: Intestinal fatty acid-binding protein
AMI: Acute mesenteric ischemia
OMI: Occlusive mesenteric ischemia
NOMI: Non-occlusive mesenteric ischemia
MODS: Multi-organ dysfunction syndrome
α-GST: α-Glutathione *S*-transferase
IMA: Ischemia-modified albumin
ABSU: Absorbance units
ELISA: Enzyme-linked immuno-sorbent assay
CT: Computed tomography
CABA: Cobalt–albumin-binding assay
ICU: Intensive care unit
LR: Likelihood ratio
QUADAS: Quality assessment of diagnostic accuracy studies
CI: Confidence interval
NPV: Negative predictive value
PPV: Positive predictive value
AAA: Abdominal aortic aneurysm
NR: Not reported
