# Supplementary material for: Diagnostic accuracy of novel serological biomarkers to detect acute mesenteric ischemia: a systematic review and meta-analysis
Source: Intern Emerg Med. 2017 May 6;12(6):821–36. doi: 10.1007/s11739-017-1668-y (PMC5559578; doi:10.1007/s11739-017-1668-y)
Supplement: Supplementary file 1 — Supplementary material 1 (DOCX 120 kb) [file 11739_2017_1668_MOESM1_ESM.docx]

|  | | |
| --- | --- | --- |
|  | **Search query** | **# hits** |
| PubMed | ((biomarker [tiab] OR “serological marker” [tiab] OR “intestinal Fatty Acid Binding Protein” [tiab] OR “intestinal fatty acid-binding protein” tiab] OR iFABP [tiab] OR I-FABP [tiab] OR d-lactate [tiab] OR “d lactate” [tiab] OR lactate [tiab] OR alpha-GST [tiab] OR “alpha-glutathione S-transferase” [tiab] OR “alpha glutathione s transferase” [tiab] OR CABA [tiab] OR “cobalt-albumin binding assay” [tiab] OR “cobalt albumin binding assay” [tiab] OR IMA [tiab] OR “ischemia-modified albumin” [tiab] OR “ischemia modified albumin” [tiab] OR “ischaemia-modified albumin” [tiab] OR “ischaemia modified albumin” [tiab] OR citrulline [tiab] OR “biological markers” [MeSH Terms] OR “fatty acid-binding proteins, intestinal specific” [MeSH Terms] OR “lactic acid” [MeSH Terms] OR “ischemia-modified albumin” [MeSH Terms] OR “glutathione S-transferase alpha” [MeSH Terms] OR “citrulline” [MeSH Terms]) AND (((Intestinal [tiab] OR Intestine [tiab] OR Mesenteric [tiab] OR Mesentery [tiab] OR Bowel [tiab] OR Gut [tiab] OR enteric [tiab] OR enteral [tiab] OR “small bowel” [tiab] OR small-bowel [tiab] OR “large bowel” [tiab] OR large-bowel [tiab] OR colon [tiab]) AND (ischemia [tiab] OR ischaemia [tiab] OR ischemic [tiab] OR ischaemic [tiab] OR necrosis [tiab] OR necrotic [tiab] OR gangrene [tiab] OR gangrenous [tiab] OR infarction [tiab] OR infarcted [tiab])) OR “abdominal compartment syndrome” [tiab] OR “Abdominal hypertension” [tiab] OR “intra-abdominal hypertension” [tiab] OR “intraabdominal hypertension” [tiab] OR “Acute abdominal disease” [tiab] OR “mesenteric arterial embolism” [tiab] OR “mesenteric embolus” [tiab] OR “mesenteric thrombus” [tiab] OR “mesenteric thromboembolism” [tiab] OR “mesenteric venous thrombosis” [tiab] OR “non-occlusive intestinal ischemia” [tiab] OR “nonocclusive intestinal ischemia” [tiab] OR “non-occlusive intestinal ischaemia” [tiab] OR “nonocclusive intestinal ischaemia” [tiab] OR “mesenteric vascular insufficiency” [tiab] OR “mesenteric infarction” [tiab] OR “bowel strangulation” [tiab] OR “strangulated bowel” [tiab] OR “bowel obstruction” [tiab] OR “ischemic colitis” [tiab] OR “ischaemic colitis” [tiab] OR “mesenteric ischemia” [MeSH Terms] OR “colitis, ischemic” [MeSH Terms] OR “intra-abdominal hypertension” [MeSH Terms] OR "intestinal obstruction" [MeSH Terms] OR "acute abdomen" [MeSH Terms]) NOT (animal [tiab] OR rat [tiab]) | 819 |
| Embase | \| **Domain** \| NOT (animal OR rat OR rats):ti,ab AND [embase]/lim \| \| --- \| --- \| \| Determinant \| 'biomarker':ab,ti OR 'serological marker':ab,ti OR 'intestinal fatty acid binding protein':ab,ti OR 'intestinal fatty acid-binding protein':ab,ti OR 'ifabp':ab,ti OR 'i-fabp':ab,ti OR 'd-lactate':ab,ti OR 'lactate':ab,ti OR 'd lactate':ab,ti OR 'alpha-gst':ab,ti OR 'alpha-glutathione s-transferase':ab,ti OR 'alpha glutathione s transferase':ab,ti OR 'caba':ab,ti OR 'cobalt-albumin binding assay':ab,ti OR 'cobalt albumin binding assay':ab,ti OR 'ima':ab,ti OR 'ischemia-modified albumin':ab,ti OR 'ischemia modified albumin':ab,ti OR 'ischaemia-modified albumin':ab,ti OR 'ischaemia modified albumin':ab,ti OR 'citrulline':ab,ti OR 'biological marker'/exp OR 'serology'/exp OR 'fatty acid binding protein 2'/exp OR 'lactic acid'/exp OR 'ischemia modified albumin'/exp OR 'glutathione transferase alpha'/exp OR 'citrulline'/exp \| \| Outcome \| ('intestinal':ab,ti OR 'intestine':ab,ti OR 'mesenteric':ab,ti OR 'mesentery':ab,ti OR 'bowel':ab,ti OR 'gut':ab,ti OR 'enteric':ab,ti OR 'enteral':ab,ti OR 'small-bowel':ab,ti OR 'large-bowel':ab,ti OR 'colon':ab,ti) **AND** ('ischemia':ab,ti OR 'ischaemia':ab,ti OR 'ischemic':ab,ti OR 'ischaemic':ab,ti OR 'necrosis':ab,ti OR 'necrotic':ab,ti OR 'gangrene':ab,ti OR 'gangrenous':ab,ti OR 'infarction':ab,ti OR 'infarcted':ab,ti)  **OR**  'abdominal compartment syndrome':ab,ti OR 'abdominal hypertension':ab,ti OR 'intra-abdominal hyprtension':ab,ti OR 'intraabdominal hyprtension':ab,ti OR 'acute abdominal disease':ab,ti OR 'mesenteric arterial embolism':ab,ti OR 'mesenteric embolus':ab,ti OR 'mesenteric thrombus':ab,ti OR 'mesenteric thromboembolism':ab,ti OR 'mesenteric venous thrombosis':ab,ti OR 'non-occlusive intestinal ischemia':ab,ti OR 'nonocclusive intestinal ischemia':ab,ti OR 'non-occlusive intestinal ischaemia':ab,ti OR 'nonocclusive intestinal ischaemia':ab,ti OR 'mesenteric vascular insufficiency':ab,ti OR 'mesenteric infarction':ab,ti OR 'bowel strangulation':ab,ti OR 'strangulated bowel':ab,ti OR 'bowel obstruction':ab,ti OR 'ischemic colitis':ab,ti OR 'ischaemic colitis':ab,ti OR 'mesenteric ischemia'/exp OR 'ischemic colitis'/exp OR 'intraabdominal hypertension'/exp OR 'intestine obstruction'/exp OR 'acute abdomen'/exp \| | 1,079 |
| The Cochrane Library | ("intestinal" OR "intestine" OR "mesenteric" OR "mesentery" OR "bowel" OR "gut" OR "enteric" OR "enteral" OR "small bowel" OR "large bowel" OR "colon") AND ("ischemia" OR "ischaemia" OR "ischemic" OR "ischaemic" OR "necrosis" OR "necrotic" OR "gangrene" OR "gangrenous" OR "infarction" OR "infarcted") OR ("abdominal compartment syndrome" OR "abdominal hypertension" OR "intra-abdominal hypertension" OR "intraabdominal hypertension" OR "acute abdominal disease" OR "mesenteric arterial embolism" OR "mesenteric embolus" OR "mesenteric thrombus" OR "mesenteric thromboembolism" OR "mesenteric venous thrombosis" OR "non-occlusive intestinal ischaemia" OR "nonocclusive intestinal ischaemia" OR "non-occlusive intestinal ischemia" OR "nonocclusive intestinal ischemia" OR "mesenteric vascular insufficiency" OR "meesenteric infarction" OR "bowel strangulation" OR "strangulated bowel" OR "bowel obstruction" OR "ischemic colitis" OR "ischaemic colitis") in Title, Abstract, Keywords and ("biomarker" OR "serological marker" OR "intestinal fatty acid binding protein" OR "intestinal fatty acid-binding protein" OR "ifabp" OR "i-fabp" OR "d-lactate" OR "d lactate" OR "lactate" OR "alpha-GST" OR "alpha-glutathione S-transferase" OR "alpha glutatione s transferase" OR "CABA" OR "cobalt-albumin binding assay" OR "cobalt albumin binding assay" OR "IMA" OR "ischemia-modified albumin" OR "ischemia modified albumin" OR "ischaemia-modified albumin" OR "ischaemia modified albumin" OR "citrulline") | 27 |
|  | | |
